# A Compact Ultra-Wideband Millimeter-Wave Four-Port Multiple-Input Multiple-Output Antenna for 5G Internet of Things Applications

**DOI:** 10.3390/s24227153

**Published:** 2024-11-07

**Authors:** Ashutosh Sharma, Sanjeev Sharma, Vikas Sharma, Girish Wadhwa, Rajeev Kumar

**Affiliations:** 1 Business School, Henan University of Science and Technology, Luoyang 471300, China; sharmaashutosh1326@gmail.com; 2 Department of Informatics, School of Computer Science, University of Petroleum and Energy Studies, Dehradun 248007, Uttarakhand, India; 3 Research Department, Rayat Bahra University, Mohali 140104, Punjab, India; sanjeev_sharma2000@yahoo.com; 4 Department of Mechanical Engineering, GLA University, Mathura 281406, Uttar Pradesh, India; vikas.sharma@gla.ac.in; 5 Amity School of Engineering and Technology, Amity University, Mohali 140306, Punjab, India; girishwadhwa12@gmail.com; 6 Graphic Era Hill University, Dehradun 248002, Uttarakhand, India; 7 Graphic Era Deemed to be University, Dehradun 248002, Uttarakhand, India; 8 Chitkara University Institute of Engineering and Technology, Chitkara University, Rajpura 140401, Punjab, India

**Keywords:** n257, n258, 261 band, ISM 24 GHz, mmWave, inset fed antennas, MIMO

## Abstract

This paper presents a compact design for a four-element multiple-input multiple-output (MIMO) antenna for millimeter-wave (mmWave) communications covering the bands of n257/n258/n261. The MIMO design covers the frequency range of 24.25–29.5 GHz, with a wide bandwidth of 5.25 GHz. The element of the MIMO antenna structure uses a single circular patch with an inset feed, and, in order to improve the reflection coefficient (S_11_), a half-disk parasitic patch is positioned on top of the circular patch. Moreover, to fine-tune the antenna’s characteristics, two vertical stubs on the extreme ends of the ground plane are introduced. For this design, a Rogers RT/Duroid 5880 substrate with ultra-thin thickness is used. After the optimization of the design, the four-port MIMO antenna attained a tiny size, with the dimensions 16.2 mm × 16.2 mm × 0.254 mm. In terms of the MIMO parameters, the ECC (Envelop Correlation coefficient) is less than 0.002 and the DG (Diversity Gain) is greater than 9.99 dB in the mentioned band, which are within the tolerance limits. Also, in spite of the very small size and the four-port configuration, the achieved isolation between the neighboring MIMO elements is less than −23.5 dB.

## 1. Introduction

Due to the enormous capabilities of 5G technologies, there has been a rapid increase in the deployment of IoT (Internet of Things) devices for a diverse range of applications, such as in smart cities, healthcare, smart manufacturing, logistics, intelligent transport systems, agriculture, etc. [1,2,3,4]. These smart IoT devices must be compact and energy-efficient, they must have low end-to-end latency, and they must be capable of transmitting large volumes of data with excellent signal quality. The surging requirement for super high data rates and bandwidth in wireless communication has driven the adoption of the mmWave (millimeter-wave) frequency band [5]. Compared to its sub-6GHz counterparts, the mmWave spectrum provides superior data rates and bandwidth. IoT-based applications, such as in industrial environments, smart health systems, autonomous vehicles, etc., require high rates. But, with the use of single antennas in the mmWave band, there is very high signal degradation due to path attenuation [6]. Therefore, mmWave antennas are more suitable for indoor short-range applications [7], such as in industrial automation, smart homes, smart healthcare, etc. To achieve optimal performance in the mmWave region and to mitigate the challenge of high attenuation, a MIMO configuration is implemented. This enables superior data rates and maintains adequate signal quality, even in adverse communication scenarios, through the use of spatial multiplexing [8,9]. Additionally, because of the shorter wavelength in mmWave, a smaller antenna size can be achieved that can support massive MIMO and beamforming [10,11]. MIMO, along with beamforming, can be implemented in IoT scenarios in Industry 4.0 or smart cities for efficient signal delivery. This enables the IoT devices to transmit large chunks of data in a shorter span of time, thus reducing the power consumption of IoT devices. The use of massive MIMO can support a large number of IoT devices, which is a primary requirement in smart cities and industrial IoT applications. In addition to supporting massive IoT deployment, the combined use of MIMO with Intelligent Reflecting Surfaces (IRSs) provides promising advantages, such as enhancement of the coverage footprint, signal quality, and reliability in densely cluttered areas [12]. MIMO antennas can also be implemented in dynamic, movable scenarios, such as in autonomous vehicles, drones, factory robots, etc. In order to attain better spatial Diversity Gain in such environments, the transmitter and/or the receiver may dynamically take positions with better channel conditions [13]. This can potentially help overcome multipath fading effects, reduce interference, enhance coverage, and improve the user experience [14].

However, because there is a common dielectric in MIMO configurations, mutual coupling due to closely placed antenna elements should be handled with diligence. There are several techniques given in the literature to minimize mutual coupling/interference, such as placing antennas elements orthogonally to each other and use of metamaterials, parasitic elements, slotted elements, neutralization lines [15], a Defected Ground Structure (DGS) [16,17], an electromagnetic band-gap (EBG), etc. [18]. However, use of these additional structures for ensuring isolation also introduces power losses and consumes more space. Among the mentioned isolation techniques, placing antenna elements orthogonally not only reduces coupling but also the space requirements.

The combined use of Ultra-Wideband (UWB) with MIMO technology finds its potential implementation in short-range communications, which can simultaneously enable enhanced data speed, wider bandwidths, decreased effects of multipath fading, and enhanced capacity [19,20]. The main parameters that need to be addressed in designing UWB-MIMO antennas are maintaining the compact size, enabling a wide bandwidth, reducing mutual coupling, etc. [21]. Several methods are proposed in the literature to design Ultra-Wideband (UWB) monopole antennas with various shapes, such as rectangular, elliptical, square, trapezoidal, circular, and other patches [22]. Alterations to these patches are achieved by etching out or through the addition of a conducting structure on the feed-plane or the ground planes so as to achieve the desired bandwidth/characteristics. The removal or addition of structures have been performed by adding stubs or by carving out slots or slits in the conducting surface, which causes perturbations in the uniform flow of the current. This can be used to enhance the bandwidth. There are various methods available in the literature using the above-mentioned techniques, which can be used to improve the bandwidth and/or reduce mutual coupling by, for example, introducing stubs in the ground/feed-plane of the L-shape [23], T-Shape [24], fork-shape [25], or stepped stub [26], ground isolating [27], etc.

In this paper, a compact Ultra-Wideband four-port MIMO antenna radiating in the 24.25–29.5 GHz range and covering the n257/n258/n261 band is proposed. The antenna structure of the four-port MIMO is very small in size, and the overall dimensions are 16.2 mm × 16.2 mm × 0.254 mm. The proposed antenna structure uses the popular inset feeding technique to control and enhance the radiation characteristicagilens of the antenna. The formation of the slit along both sides of the feedline provides junction capacitance that can modify the resonance frequency of the antenna. Also, the dimensions of the inset length and the inset width of the slit can be varied to influence the resonant frequency and other characteristics [28].

The proposed four-port MIMO antenna has very small dimensions. In spite of the four-port configuration and the very small size, the proposed MIMO antenna offers acceptable isolation of >23 dB. The presented antenna has a simple planar design; it avoids any complicated structures, such as vias or multiple layers, and it uses an ultra-thin Rogers substrate of 0.254 mm, which makes it easy to fabricate. The MIMO antenna delivers a wide bandwidth of about 5.25 GHz and a peak gain of about 6 dB. All of these benefits make the proposed structure suitable for application using an n257/n258/n261 band.

In Section 2, we discuss the design aspect of the proposed antenna. In Section 3, we describe the details of how the step-wise development of the antenna design was undertaken. Section 4 deals with how the dimensional parameters of the antenna are altered to obtain optimized results.

## 2. Antenna Design

The antenna is easy to fabricate on Rogers RT Duroid 5880 substrate due to its simple and symmetrical structure, but it requires precise alignment of the ground and the feed-plane.

**Figure 1:**
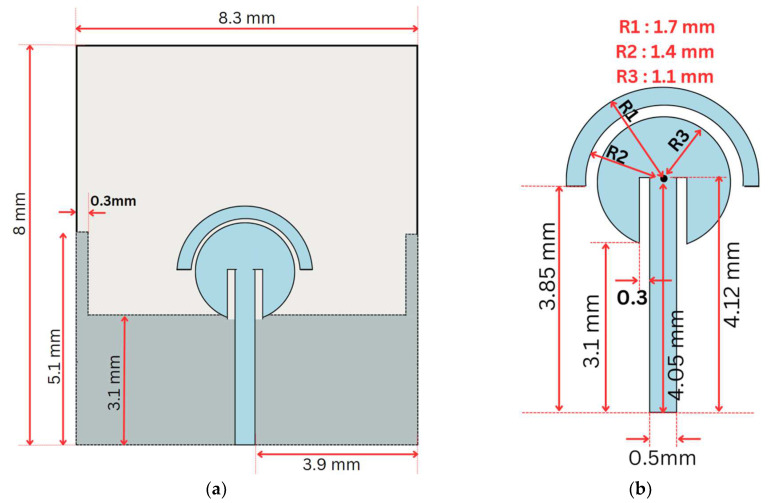
Design structure of single-element antenna. (**a**) Single element of MIMO antenna design. (**b**) Feedline with circular and semi-circular patch.

Feed-Plane: The single-element structural design of the MIMO antenna is shown in Figure 1, which comprises a circular patch with a radius of 1.1 mm, and this patch is covered from the top by another half-disk-shaped parasitic structure with an outer radius of 1.7 mm and an inner radius of 1.4 mm. There is a separation of 0.3 mm between the outer disk and the inner circle. Additionally, there are two slits with the dimensions 1.02 mm × 0.3 mm; each is placed on either side of the feedline. The circular patch and the parasitic disk are fed by the feedline, which has the dimensions 4.12 mm × 0.5 mm, which is placed in the center of the overall antenna structure, as shown in Figure 1b.

Ground Plane: The ground plane consists of a rectangular patch measuring 8.3 mm× 3.1 mm, which is aligned just below the circular feed structure, as shown in Figure 1a. Two thin, rectangular-shaped, protruding stubs measuring 2 mm × 0.3 mm are positioned on the extreme edges of the ground plane.

**Figure 2:**
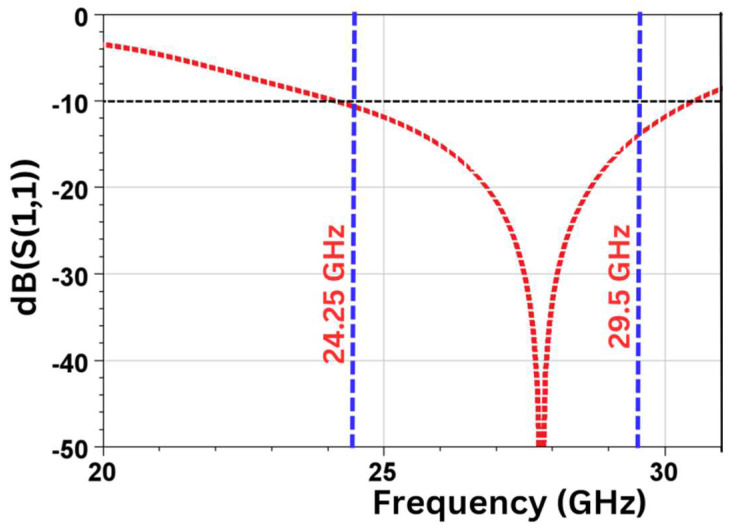
Reflection coefficient (S_11_) vs. frequency for single-element antenna.

Below, in Figure 2, the simulated result of the reflection coefficient (S_11_) is depicted for the single-element antenna. The simulation results are obtained by using Ansys HFSS software (2024 R1). It is observed that based on the −10 dB impedance bandwidth reference, excellent values of S_11_ are achieved in the frequency range of 24.25 to 29.50 GHz, resulting in a bandwidth of 5.25 GHz. This completely covers the n257/n258/n261 band frequency of FR2.

## 3. Evolution

Although the final structural design is achieved after numerous iterations through an optimization process, for clarity, we have summarized the evolution process in three simple steps. To achieve the final design, variations in the size and shape of the structural components are made at each step. The step-wise summary of the activity is given below.

**Figure 3:**
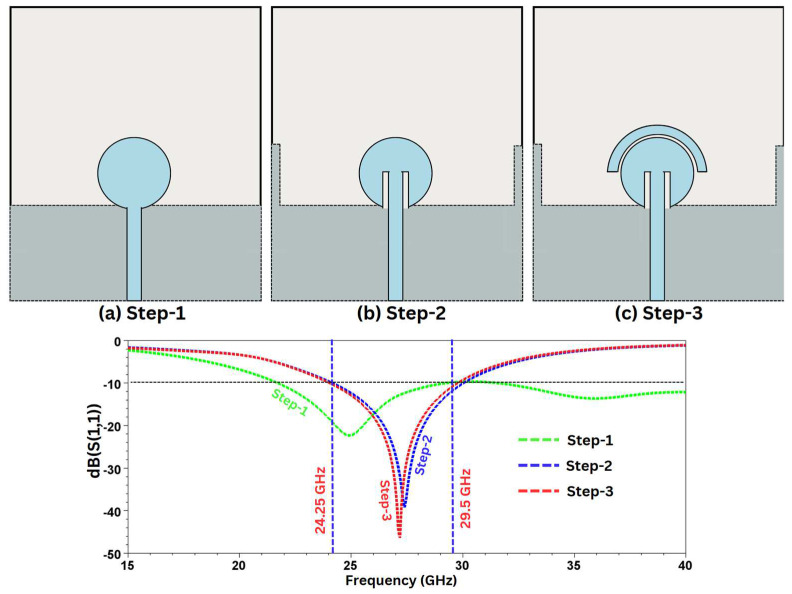
Step-wise antenna design.

Step 1: Initially, as depicted in Figure 3a, a simple structure is created with a circular patch, which is fed by a feedline. The rectangular-shaped ground plate is aligned at the level just below the circular disk of the feed. The structure generates wideband characteristics; however, the S_11_ is poor, and the bandwidth is much larger than we require.

Initially, the radius ‘a’ of the circular patch, which can directly impact the resonant frequency, can be calculated using the formula below [29].

$$a=\frac{F}{\sqrt{1+\frac{2h}{\pi .ℇr.F\;}\left[\ln\left(\frac{\mathrm{F\pi}}{2h}\right)+1.7726\right]}}$$

where $F=\frac{8.791\times 10^{9}}{f_{r}\;\sqrt{{ℇ}_{r}}}$, h is the substrate height and ℇr is the substrate’s dielectric constant.

Due to fringing effects, the dimensions of the circular patch are modified, and the effective radius (a_eff_) of the circular patch can be calculated using the equation below.

$$a_{eff}=a\sqrt{1+q},\;\mathrm{where}\;q=\frac{2h}{\pi .ℇr.a\;}\left[\ln\left(\frac{a.\pi }{2h}\right)+1.7726\right]$$

Step 2: In this step, two alterations are made. Firstly, we place two protruding stubs on the extreme edges of the ground plane. This helps in increasing the effective length of the radiating surface, and it also reduces the overall size of the design. Secondly, in the feed-plane, two slits are etched out on both sides of the feedline, as displayed in Figure 3b. With these changes, the antenna starts resonating at the desired frequency.

Step 3: To further improve upon the return loss, a half-disk-shaped parasitic structure is placed on top of the circular patch, as shown in Figure 3c.

**Figure 4:**
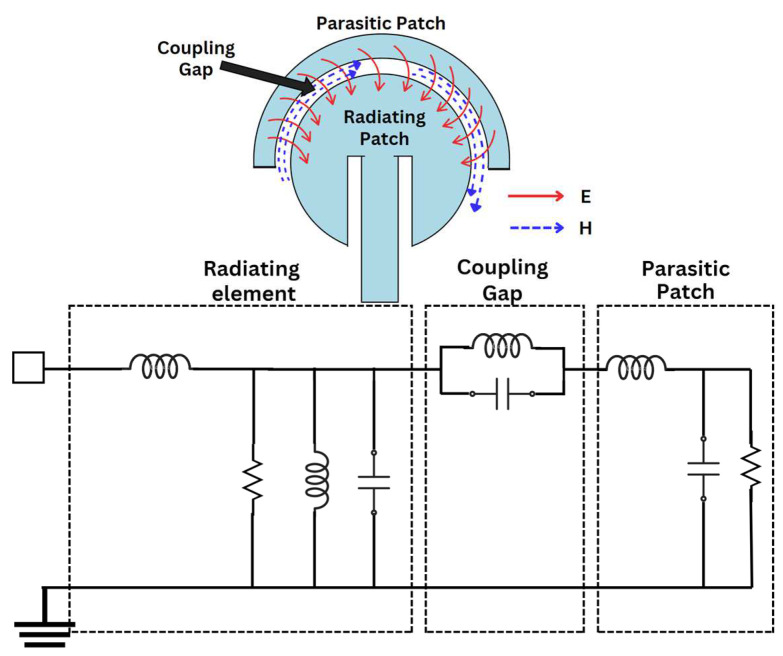
Radiating and parasitic patch equivalent circuit.

**Figure 5:**
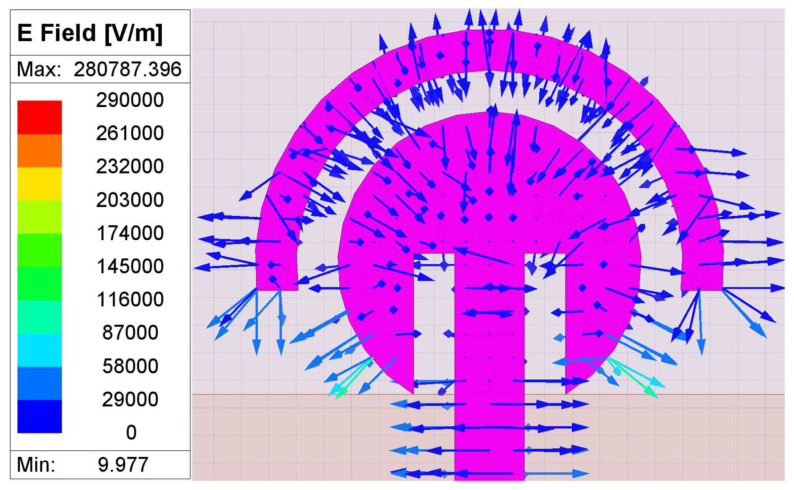
E field distribution analysis between radiating and parasitic patch.

A parasitic element is a conductive element that acts like a passive radiator and that can be used to improve the antenna’s parameters, such as the bandwidth and the reflection coefficients. In ref. [30], an improvement in the bandwidth and the stable radiation pattern is suggested by the use of a parasitic element. Also, ref. [31] suggests an improvement in both the bandwidth and impedance matching. In the proposed structure, the addition of a parasitic patch has resulted in a better reflection coefficient, which is depicted in Figure 3c. Moreover, the introduction of a parasitic structure forms guided E and H fields between the radiating and the parasitic patch due to the creation of a coupling effect in the gap, as illustrated in Figure 4. This leads to an enhancement of characteristics of the design, such as the reflection coefficients (due to better impedance matching). The guided E and H fields in the coupling gap can be represented by an equivalent circuit where L and C are placed in a parallel combination [32,33]. The distribution of the E field between the radiating circular patch and the parasitic structure is represented in Figure 5.

## 4. Parametric Analysis

The optimization task has been performed by altering the dimensions of the structures in the antenna design. Different dimensions of the parameters have different levels of impact on the radiation characteristics of the design. In this section, those parameters of the design will be discussed that have a significant effect on the antenna’s characteristics.

**Figure 6:**
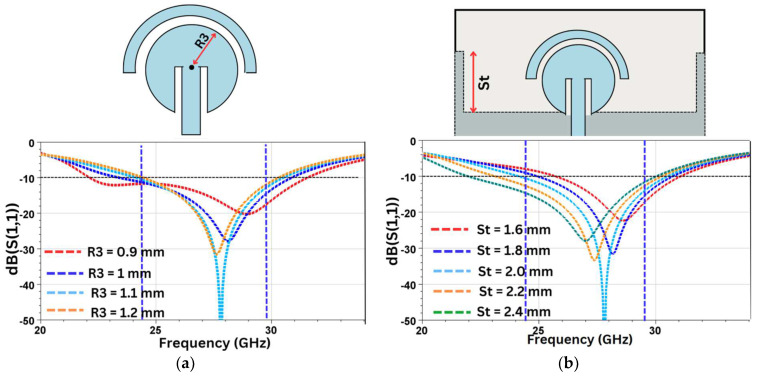
Optimization of S_11_ and resonant frequency using (**a**) R3 and (**b**) St.

Radius of the feed-plane circle (R3): The change in the values of the radius (R3) of the circular structure along with half-disk parasitic cover changes both the return loss and resonating frequency. Figure 6a depicts these variations with the change in the radius R3. As can be seen, an R3 value of 1.1 mm gives the optimal value of S_11_.

Length of stub in the ground plane (St): As the length of the stub is increased, the effective length of the radiating surface also increases, which causes the resonant frequency to shift to lower values. However, optimal results are attained with St = 2.00 mm, as seen in Figure 6b.

**Figure 7:**
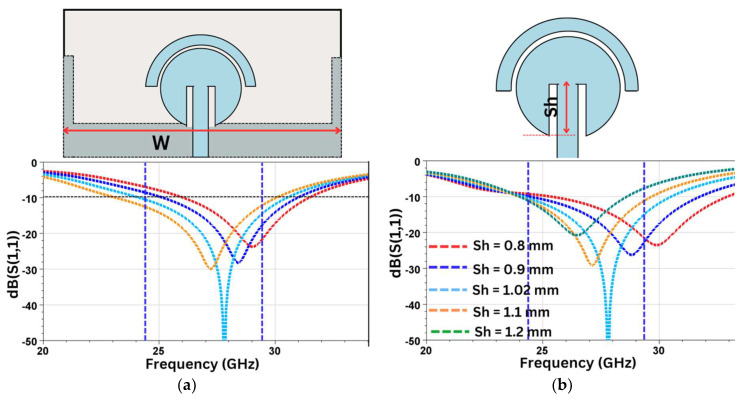
Optimization of S_11_ with (**a**) W and (**b**) Sh.

Width of ground plane surface (W): The radiating effective length is also increased by increasing the width of the ground plane (W). The optimum results for S_11_ are achieved with W = 8.3 mm, as shown in Figure 7a.

Length of inset feed (slit on both sides of the feedline, Sh): The slit placed on either side of the feedline has a strong influence on the radiation characteristics of the design. This technique can be used to improve and fine-tune the antenna’s characteristics. As seen in Figure 7b, a variation in the length of Sh influences the resonant frequency, S_11_, and the bandwidth. The optimum value for our configuration is achieved at Sh = 1.02 mm.

Based on the above discussion, we can clearly infer that the proposed structure can be made to resonate over a wide range of frequencies with minor alterations of the dimensional parameters, like R3, St, W, and Sh.

## 5. MIMO Antenna Design

**Figure 8:**
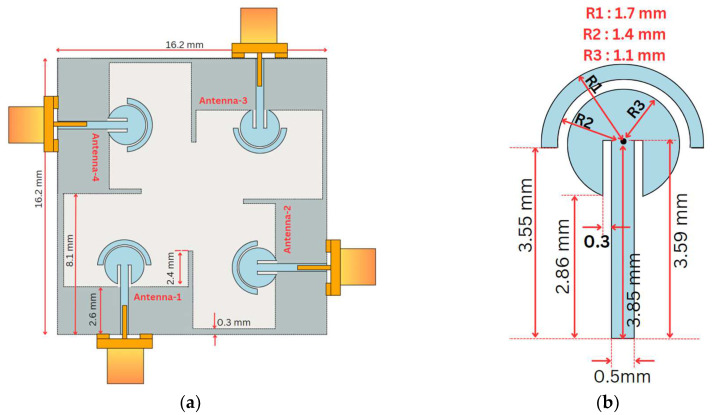
MIMO antenna design. (**a**) Top view, (**b**) modified feedline.

The single-element antenna design described above is used to develop a four-element MIMO antenna so as to attain both the high channel capacity and data rates in the mmWave frequency. As depicted in Figure 8, the proposed MIMO antenna has four antennas placed orthogonally (perpendicular) to each other to achieve adequate isolation between them, as in ref. [34].

Each antenna element has a separate ground plane with a separate set of protruding stubs on the edge of each ground plane. The dimensions of these two protruding stubs, St, and the height of the ground plane have been updated to 2.4 mm and 2.6 mm, respectively, as shown in Figure 6a. Also, the dimensions of the feedline have been updated for each individual MIMO element so as to fine-tune the antenna’s performance in MIMO configurations. The optimized size for the proposed MIMO antenna structure is 16.2 mm × 16.2 mm.

**Figure 9:**
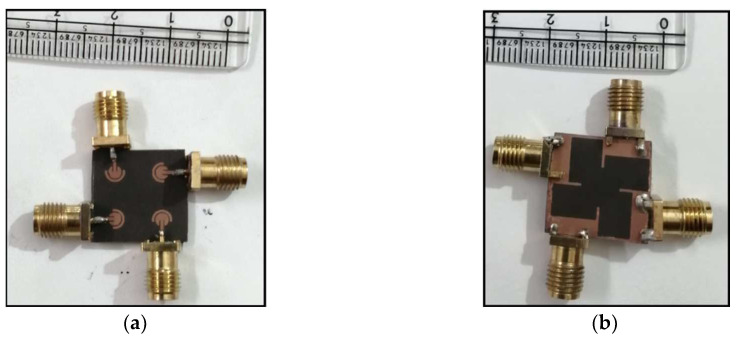
Fabricated prototype of the proposed antenna. (**a**) Feed-plane, (**b**) ground plane.

In order to have adequate isolation in the MIMO configuration, the separation between the individual MIMO elements has been optimally increased. In addition, a 0.3 mm wide connecting line in the ground plane has been added between the individual MIMO elements so as to have a co-surfaced ground plane [35]. Figure 9 shows the fabricated prototype of the proposed antenna.

## 6. Results and Discussion

### 6.1. S-Parameters

**Figure 10:**
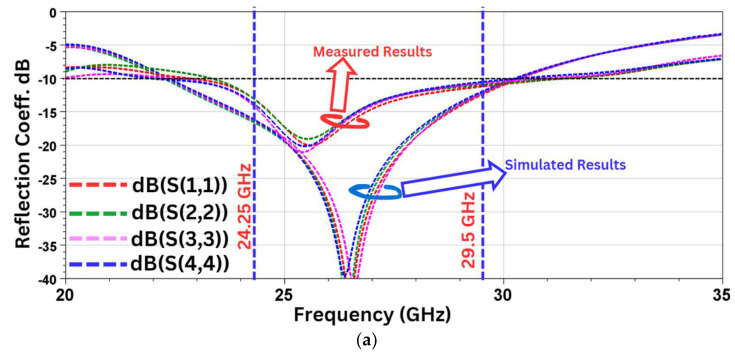

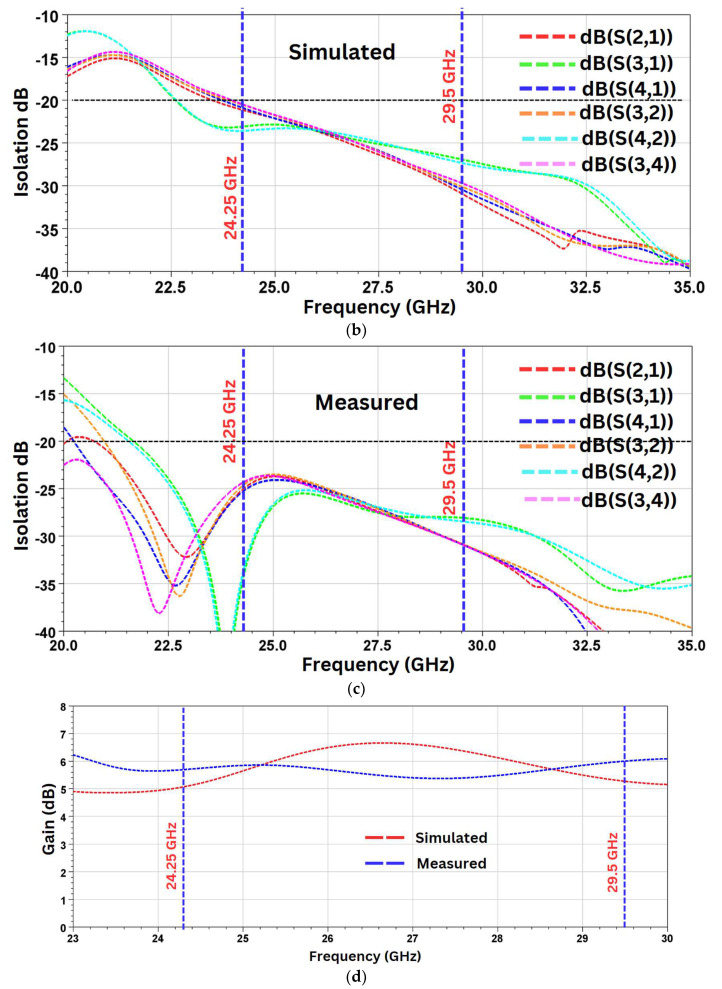
Results. (**a**) Reflection coefficients measured and simulated, (**b**) simulated isolation, (**c**) measured isolation, (**d**) gain vs. frequency plot.

The simulated and measured results with all four radiating elements of the MIMO antenna are shown in Figure 8. The measurement has been performed by using the Agilent Vector Network Analyzer N5247A under an anechoic chamber measuring 10 ft (length) × 12 ft (breadth) × 9 ft (height). As seen in Figure 10a, there is a shift in the measured operating frequency compared to the simulated results. The measured result shows that Antenna-1 radiates between 22.8 and 31.5 GHz, Antenna-2 between 23.8 and 31.5 GHz, Antenna-3 between 22.4 and 31.1 GHz, and Antenna-4 between 22.0 and 30.5 GHz. The shift in both the S_11_ and the radiating frequency may be attributed to the large size of the SMA connectors compared to the small size of the antenna. Also, due to the minute size of the antenna, any small deviation in the dimensions or the alignment during physical antenna fabrication impacts the output characteristics. But, in spite of the shift in frequency in the measured results, the required frequency band from 24.25 to 29.5 GHz is completely covered.

**Figure 11:**
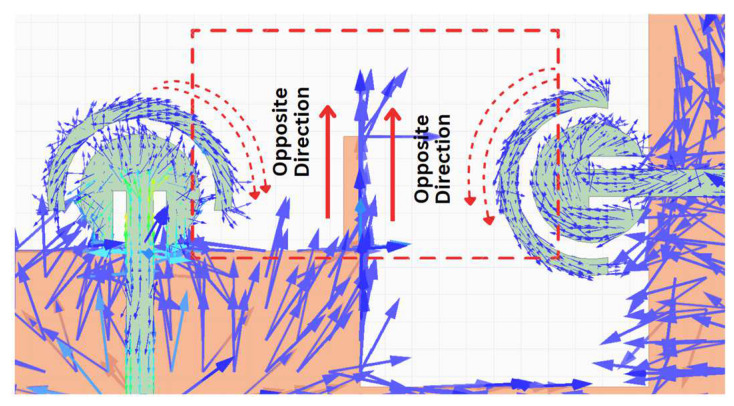
Decoupling due to protruding stubs.

Also, it can be seen from Figure 10b,c that the measured isolation of the adjacent antennas for the desired bandwidth is less than −23.5 dB. Even though the edge-to-edge distance between the radiating elements is just 4.9 mm, which is 0.4λ_0_ at 28 GHz, the measured coupling is lower than −23 dB. The low value of coupling between the MIMO elements is partially due to the orthogonal placement of the antenna elements with respect to each other, which not only helps to reduce the overall dimensions but also to achieve low mutual coupling. In addition, as shown in Figure 11, the protruding stubs on the edge of the ground plane also induce a current with a direction opposite to that of the monopole semi-circle, which further reduces the value of mutual coupling [36].

The simulated and measured gain vs. the frequency plot is shown in Figure 10d.

### 6.2. Radiation Characteristics

**Figure 12:**
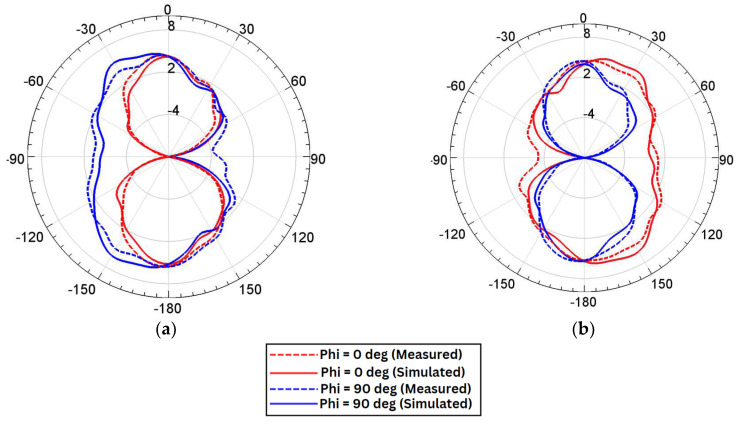

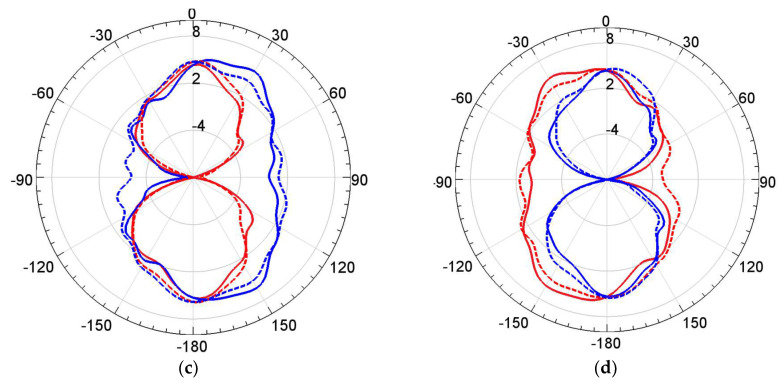
Radiation characteristics. (**a**) Antenna-1. (**b**) Antenna-2. (**c**) Antenna-3. (**d**) Antenna-4.

The simulated and measured results of the far-field radiation pattern of the proposed four-element MIMO antenna are as shown in Figure 12a–d. The radiation pattern is captured by keeping the frequency at 28 GHz. The gain characteristics of each antenna are presented for Φ = 0° and Φ = 90°.

### 6.3. MIMO Parameters

In the MIMO antenna design, the diversity parameters play a vital role in determining the MIMO performance. Among these parameters, the Diversity Gain (DG), the Envelop Correlation coefficient (ECC), and the mean effective gain (MEG) are important. The ECC is used to determine the extent to which the MIMO antenna elements are isolated. Ideally, the value of ECC should be near to zero, but, in practical applications, a value less than 0.05 is considered acceptable. The value of the ECC for the MIMO antenna can be calculated based on the S-parameters using the following formula [37]:

$$ECC=\frac{{\left|Sjj*Sji+Sij*Sii\right|}^{2}}{{\left(1-|Sjj\right|}^{2}-{\left|Sji\right|}^{2}){\left(1-|Sij\right|}^{2}-{\left|Sii\right|}^{2})}$$

where *S*_*ii*_ & *S*_*jj*_ are reflection coefficients and *S*_*ji*_ and *S*_*ij*_ are the transmission coefficients. ECC can also be calculated from 3D radiation patterns, which can be measured in an anechoic chamber using the equation below [38].

$$\left|\rho \right|=\frac{{\left|\oint E_{i}\cdot E_{j}^{*}\;d\Omega \right|}^{2}}{\left|\oint E_{i}\cdot E_{i}^{*}\;d\Omega \times \oint E_{j}\cdot E_{j}^{*}\;d\Omega \right|\prime }$$

where E is the far-field radiation pattern (E field), which is received by the measurement probe, and Ω=sin⁡θ dθd∅ is the beam solid angle.

**Figure 13:**
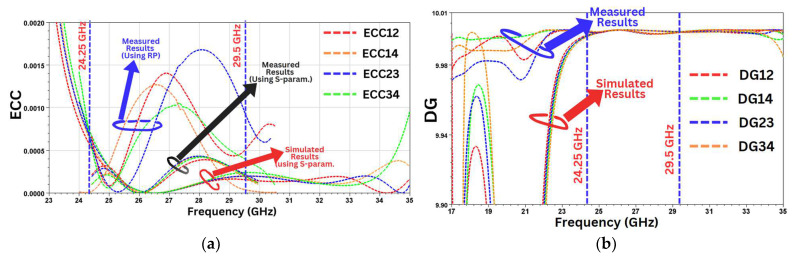
(**a**) Envelop Correlation coefficient. (**b**) Diversity Gain.

We can see from Figure 13a that the measured ECC value is less than 0.002 in the desired frequency band (24.25–29.5 GHz).

The Diversity Gain (DG) is calculated using the formula mentioned in [39], which is stated below.

$$DG=10\sqrt{\left(1-{ECC}^{2}\right)}$$

As shown in Figure 13b, the measured value attained for DG is greater than 9.99 dB.

The total active reflection coefficient (TARC) is another important MIMO parameter used to qualify the extent of mutual coupling between the ports. For the MIMO antenna array, TARC indicates the ratio of the reflected power with the incident power.

What follows is the equation for the calculation of the TARC:

$$TARC=\frac{\sqrt{{\left(|Sii+Sij\right|}^{2})+({\left|Sji+Sjj\right|}^{2})}}{\sqrt{2\;}}$$

The Channel Capacity Loss (CCL) is yet another key MIMO performance indicator. Calculation of the CCL is performed by using the equation below.

$$CCL=-{log}_{2}{(\Psi }^{R})$$

where $\Psi^{R}$ is the receiver antenna’s correlation matrix, which is given below.

$$\Psi^{R}=\begin{array}{cc} \Psi_{ii} & \Psi_{ij}\\ \Psi_{ji} & \Psi_{jj} \end{array}$$

$$\Psi_{ii}=1-{\left(|S_{ii}\right|}^{2}+{\left|S_{ij}\right|}^{2}),\;\Psi_{ij}=-(S_{ii}*S_{ij}+S_{ji}*S_{jj}),$$

$$\Psi_{ji}=-(S_{jj}*S_{ji}+S_{ij}*S_{ii}),\;\Psi_{jj}=1-{\left(|S_{jj}\right|}^{2}+{\left|S_{ji}\right|}^{2})$$

The value of the CCL should be lower than 0.4 bits/s/Hz.

**Figure 14:**
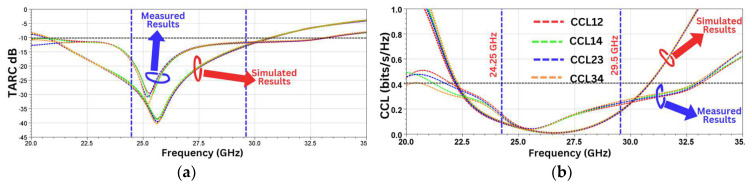
(**a**) TARC. (**b**) CCL.

Figure 14a,b show the simulated and measured values of the TARC and the CCL, respectively, which are well within the thresholds.

### 6.4. Surface Current

**Figure 15:**
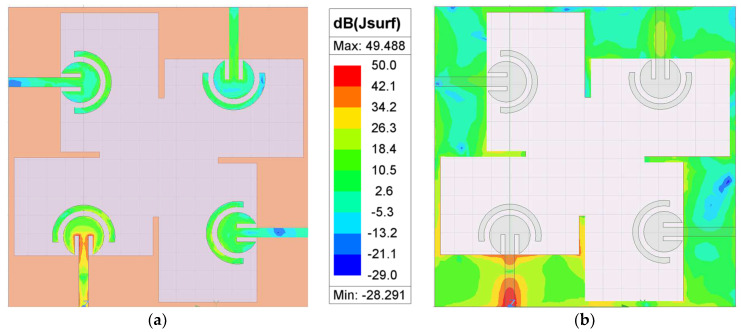
Surface current for Antenna-1 at 27 GHz. (**a**) Feed-plane (top view). (**b**) Ground plane (top view).

The induced surface current at 27 GHz for Antenna-1 is shown in Figure 15. As it can be seen from Figure 15b, a large current is induced along the length of W and St on the ground plane. This is in line with our earlier discussion in Section 4, where it is noted that there is a significant change in the resonant frequency when the length of W and St is changed. Additionally, as seen in Figure 15a, a large current is inducted along slits placed on both sides of the feedline. This is again in line with our previous discussion, where there is large change in the radiation characteristics when the length of Sh (the slit length) is altered. So, we can clearly infer that the length of the ground plane and the slit length have a significant role to play in achieving the antenna’s response. The surface current for only one antenna element is shown, as other antennas also follow a similar pattern.

### 6.5. Comparison with Related Work

As seen in the comparative table (Table 1), our work offers the smallest dimensional configuration. Also, the proposed work has a four-port MIMO configuration, which is the highest among other mentioned studies. Despite the smaller size and the four-port configuration of our work, an acceptable value isolation of >23 dB is achieved. Refs. [40,41], which describe a two-port and a three-port configuration, respectively, offer good isolation, but the size is large and the bandwidth is relatively small. Ref. [42] offers the highest gain; however, it uses two dielectric layers, which not only increases the dimensions but is also comparatively difficult to fabricate. The proposed antenna is a simple planar configuration, and it avoids vias or SIWs, which makes it easy to fabricate.

## 7. Conclusions

In this paper, we have furnished a small-sized antenna covering the n257/n258/n261 band radiating in a frequency range from 24.25 to 29.5 GHz with a bandwidth of 5.25 GHz. The antenna also covers the ISM24GHz band. Firstly, the characteristics of the single-element antenna were described, and, later on, the design was extended to a four-port MIMO antenna. Very small overall dimensions of 16.2 mm × 16.2 mm × 0.254 mm are achieved for the proposed four-port antenna. With regard to the MIMO parameters, the ECC is less than 0.002, and the DG is greater than 9.99 dB, and these parameters, as discussed in relation to the mentioned band, are within the tolerance limits. The isolation between the neighboring MIMO elements is also less than −23.5 dB.

## Figures and Tables

**Table 1:** Comparison between related studies.

Ref.	Structure Type	Center Freq./Band	Bandwidth (GHz)	Size (mm)	No of Ports	Peak Gain (dBi)	DG (dB)	ECC	Min Isolation
[40]	Air-filled slot	28 GHz	0.4	33 × 27.5 × 0.76	2	6.9	9.9998	0.001	30
[41]	SIW-based	28 GHz	0.55	26 × 26 × 0.76	3	4.71	-	<0.002	>30
[42]	Fabry–Perot (2-layer)	28 GHz	7	19 ×19 × 7.608	4	14.1	-	<0.008	>25
[43]	Defected ground array	28 GHz	4.1	30 × 35.5 × 0.76	4	8.02	>9.96	<0.01	17
[44]	Dielectric resonator	28 GHz	0.85	20 × 20 × 2.54	2	8	9.9	0.13	24
[45]	Coplanar feed patch	n257/n258/n261 (23–30.5 GHz)	7.5	26 × 5 × 1.524	4	9.76	-	-	>16
Our Work	Circular Patch	28 GHz	5.25	16.2 × 16.2 × 0.254	4	6.09	>9.99	<0.002	>23.5

## Data Availability

No new data were created or analyzed in this study. Data sharing is not applicable to this article.
